# *JAG2*: A Potential Biomarker for Microtia Identified by Integrated RNA Transcriptome Analysis

**DOI:** 10.2174/0113892029311725240911065539

**Published:** 2024-09-25

**Authors:** Xu Wu, Yaoyao Fu, Jing Ma, Chenlong Li, Tianyu Zhang, Aijuan He

**Affiliations:** 1 Department of Facial Plastic and Reconstructive Surgery, Eye & ENT Hospital, Fudan University, Shanghai, China;; 2 ENT Institute, Eye & ENT Hospital, Fudan University, Shanghai, China;; 3 NHC Key Laboratory of Hearing Medicine (Fudan University), Shanghai, 200031, China

**Keywords:** Chondrocyte, microtia, RNA transcriptome, bioinformatics, *JAG2*, maxillofacial deformity

## Abstract

**Introduction:**

Microtia, a prevalent congenital maxillofacial deformity, significantly impacts the physical and psychological health of children. Its etiology, especially in non-syndromic cases, remains a complex and partially understood domain, complicating etiological treatment. Recent studies pointed to a genetic predisposition in non-syndromic microtia, yet research on susceptible or pathogenic genes is limited.

**Objectives:**

This study focused on identifying key biomarker genes in microtia cartilage to elucidate pathogenesis and assist in prenatal diagnosis.

**Methods:**

We first collated two bulk transcriptome datasets from the GEO database, followed by functional enrichment analysis and Weighted Gene Co-expression Network Analysis (WGCNA) to pinpoint differentially expressed genes (DEGs) and gene modules. The subsequent intersection of DEGs with WGCNA modules, aided by support vector machine-recursive feature elimination (SVM-RFE) and protein-protein interaction (PPI) networks, predicted potential susceptibility genes for microtia. Finally, we integrated bulk RNA sequencing with single-cell data *via* the “scissor” R package and further validated it with Real-time PCR and immunofluorescence.

**Results:**

We identified *JAG2* as a prominent biomarker for microtia, evidenced by its significant upregulation in microtia cartilage.

**Conclusion:**

Our findings implicate *JAG2* in microtia development and suggest its role in chondrocyte maturation and differentiation through Notch signaling pathway activation, shedding light on the potential pathogenesis of microtia.

## 1 INTRODUCTION

Congenital microtia is a prevalent congenital maxillofacial deformity with a global incidence ranging from 0.84 to 17.4 per 10,000 individuals [1]. In China, the incidence of congenital microtia is approximately 0.81 to 3.06 cases per 10,000 newborns [2]. Characterized by underdeveloped or completely absent ears, often accompanied by misaligned jaws and chin, microtia not only affects physical appearance but also leads to significant social and psychological challenges [3-5]. It is categorized into four types based on severity and into syndromic or non-syndromic types, with the latter representing 73% of cases [2, 6]. The etiology of non-syndromic microtia, though multifaceted, is not completely understood, with increasing evidence pointing to genetic factors. Identified genes in signaling pathways include FGFR2 [7], BMP5 [8, 9], HOXA2 [10, 11] *etc*. However, the phenotypic variability and complex etiology of non-syndromic microtia obscure many potential susceptibility or pathogenic genes, complicating both etiological treatment and early diagnosis.

The rapid advancement of high-throughput sequencing technologies, notably RNA transcriptome and single-cell RNA sequencing, have opened new avenues for understanding the etiology of microtia and its clinical implications [12]. Bioinformatics analyses leveraging RNA sequencing data have been instrumental in exploring the genetic underpinnings of microtia. However, limitations, such as small sample sizes in prior studies and the inadequacy of bulk RNA-seq in capturing the disease's complex etiology, have been noted. An integrated approach combining expanded RNA transcriptome datasets and merging bulk RNA-seq with single-cell RNA sequencing data could offer a more comprehensive genetic insight into microtia, potentially leading to more accurate and clinically relevant findings [13, 14].

To identify potential pathogenic genes and abnormal signaling pathways in congenital microtia, we conducted a comprehensive analysis integrating bulk and single-cell transcriptomic data. We began by merging two bulk transcriptome datasets (GSE227119 and GSE225225) from the Gene Expression Omnibus (GEO) database, encompassing a total of 13 microtia and 8 normal samples. Weighted gene co-expression network analysis (WGCNA) was then employed to pinpoint differentially expressed genes (DEGs) and genes exhibiting significant associations with clinical phenotypes. To further refine candidate diagnostic biomarkers, we intersected the DEGs with WGCNA results. We subjected them to analysis using support vector machine-recursive feature elimination (SVM-RFE) and protein-protein interaction network (PPI) analysis. Subsequently, we leveraged the R package “scissor” to integrate bulk and single-cell data (GSE179135), validating these candidate genes. Real-time PCR and immunofluorescence techniques were additionally employed to elucidate the potential involvement of these genes in microtia pathogenesis.

## 2 MATERIALS AND METHODS

### 2.1 Data Collection

We sourced one RNA transcriptome dataset, GSE227119, from the GEO (Gene Expression Omnibus) database. This dataset included 9 samples: 3 from normal auricular cartilage, 3 from mildly deformed auricular cartilage (MIC II, Microtia grade two), and 3 from severely deformed auricular cartilage (MIC III, Microtia grade three). Additionally, we obtained another RNA transcriptome dataset, GSE225225, from the corresponding author [15]. This dataset comprised 15 samples, with 10 representing normal auricular cartilage and 5 characterized by MIC III. For rigorous analysis, we selectively included data only from the normal and MIC III groups. Furthermore, the single-cell transcriptome expression dataset GSE179135, also retrieved from the GEO database, encompassed 9 samples, comprising 6 from normal auricular cartilage and 3 from MIC III cartilage.

The two bulk datasets were merged using the R software package “sva,” the batch effect was removed using the combat function, and data adjustment was performed using “FDR” to obtain the final merged dataset. The merged data included 13 normal samples and 8 microtia samples. Fig. (**1**) shows the whole flowchart of this study.

### 2.2 DEGs Identification

DEGs from the merged data were identified using the R package “DESeq2”, applying cutoff criteria of adjusted *P*-value < 0.05 and log2FoldChange > (mean∣log_2_FoldChange∣ + 2*sd∣log_2_FoldChange∣).

### 2.3 Identification of Gene Modules by WGCNA

WGCNA was performed to identify the correlation to clinical phenotypes (Microtia or Normal) of module’s gene expression using the R package “WGCNA.” Genes with similar expression patterns were selected based on the soft-threshold power. To reduce false correlations and calculate the degree of association between genes, we utilized the topological overlap matrix (TOM). Subsequently, genes were grouped into various modules according to TOM-based dissimilarity measurements. Each module 's gene expression profile was represented by its module eigengene (ME). Pearson 's correlation was employed to assess the relationship between ME and clinical phenotype, thereby evaluating each module’s association with clinical phenotype. The CC (Correlation Coefficient) and *P*-values of each module in microtia and normal groups are presented in the center of the panels. The closer the CC is to 1, the higher its relevance is.

### 2.4 GO, KEGG and GSEA Enrichment Analysis

To gain further insights, Gene Ontology (GO) functional annotation and Kyoto Encyclopedia of Genes and Genomes (KEGG) pathway enrichment analysis of vital genes were performed using R package the “clusterProfile” [16]. Gene Set Enrichment Analysis (GSEA) was conducted using the R package “GSEA.”

### 2.5 Feature Gene Selection by SVM-RFE Algorithms

SVM-RFE is a machine learning algorithm for feature selection and model building, which is an extension of support vector machines. Here, we used the R package “Caret” to select hyperparameters for all classifiers using 10-fold cross-validation for the training dataset. We also identified the diagnostic value of genes with higher discriminative power by the “e1071” package.

### 2.6 PPI Network and Hub Genes Screening

PPI network analysis was performed using the online database STRING44 with the confidence of parameter (interaction score > 0.400). Hub genes were screened by “cytoHubba” in the software “Cytoscape.”

### 2.7 Real-time PCR Analysis and Immunofluorescence Evaluation

RT-PCR and immunofluorescence evaluation were further performed in this study. Ethical approval for the study was obtained from the medical ethics committee of the Eye & ENT Hospital of Fudan University, and all participants provided written informed consent prior to their inclusion in the study. Microtia auricular cartilage specimens were obtained from 4 microtia patients (9-12 years of age) attending the Eye & ENT Hospital of Fudan University (Shanghai, P.R. China). Normal auricular cartilage specimens were obtained from 4 patients who underwent surgery for otitis media at the Eye & ENT Hospital of Fudan University (Shanghai, P.R. China). Demographic characteristics are shown in Table **1**.

The cartilage tissue from each specimen was dissected to remove fibrous tissue. Subsequently, chondrocytes were isolated by mincing the cartilage specimens and digesting them with 0.15% collagenase (Sigma‒Aldrich, St. Louis, MO, USA), following previously described methods [17-19].

Chondrocytes from microtia patients and normal auricular cartilage were collected for RT-PCR analysis and immunofluorescence staining. RNA was first extracted and then reverse-transcribed into single-stranded cDNA. Real-time PCR analysis was conducted following previously described protocols [17, 20]. The primer sequence of *JAG2* for RT-PCR was 5′-AGCCATGCCTTAACGCTTTT-3′(forward), 5′-CACACACTGGTACCCGTTCA-3′ (reverse).

After fixing on glass slides and washed with phosphate-buffered saline (PBS) prior to fluorescent staining, chondrocytes were incubated overnight at 4 °C with primary JAG2 antibody (PA5-111319, Invitrogen, USA), followed by subsequent incubation with secondary antibodies conjugated with fluorophores or horseradish peroxidase at room temperature for 1 h in the dark. DAPI was employed for nuclear staining. The laser confocal microscope was used to visualize cellular fluorescence.

### 2.8 Single-cell Transcriptome Data Analysis

For the analysis of GSE179135 single-cell transcriptome data, we employed Seurat [21] for normalization, scaling, and clustering of cells to obtain 3 distinct clusters. Single cells were chosen using selection criteria of 2000< nFeature_RNA<6000, nCount_RNA>8000, and percent. MT<5% to remove doublets and nonviable cells. The filtered gene-barcode matrix was normalized using the “LogNormalize” method *via* the “NormalizeData” function. The top 2000 variable genes were identified with the “FindVariableFeatures” function using the “vst” process, and PCA was conducted for dimensionality reduction. To mitigate batch effects among samples, we applied the “Harmony” [22] R package. Following this step, functions such as “FindNeighbors,” “FindClusters,” and “runUMAP” was utilized to visualize all cell types on a two-dimensional map. To assess differential gene expression between groups, we employed the Kruskal‒Wallis test.

To identify cell populations associated with clinical phenotypes in single-cell transcriptome data, we employed the R package “Scissor” to integrate bulk data into single-cell data [23]. Following this step, the function “Findmarker” was used to analyze differentially expressed genes between scissor- and scissor+ cells.

### 2.9 Package Versions

Rstudio_4.2.2;ClusterGVis_0.1.1;monocle_2.29.0;Seurat_4.3.0.1;DEseq2_1.40.2;Scissor_2.0.0;Sva_3.48.0;WGCNA_1.72.1;e1071_1.7.13;kernlab_0.9.32;caret_6.0.94;clusterProfiler_4.9.1;Cytoscape_3.10.0;GSEA_3.15; sva_3.48.0.

## 3 RESULTS

### 3.1 DEGs between Normal and Microtia Samples

Prior to batch effect removal using the R package “sva,” the Principal Component Analysis (PCA) plot disclosed a notable principal component disparity between the two datasets, as depicted in Fig. (**2A**) (before). The batch effect was effectively mitigated post “sva” application in the merged dataset, evident in Fig. (**2B**) (after). Analysis of this adjusted data led to the identification of 669 DEGs. Within this subset, 396 genes were found to be significantly up-regulated, while 273 genes were significantly down-regulated. These DEGs are graphically represented in the volcano plot shown in Fig. (**2C**).

### 3.2 Key Gene Modules Associated with Microtia

In our endeavor to pinpoint pivotal gene modules associated with microtia, we executed a WGCNA on the merged dataset. This analysis revealed 9 distinct gene modules, as illustrated in Figs. (**2D** and **2E**). Delving into the module-trait relationships, depicted in Fig. (**2F**), we observed that the green gene module exhibited a substantial negative correlation with microtia (*P*-value = 1e-04, Correlation Coefficient (CC) = 0.77). In contrast, the red module demonstrated a significant positive correlation (*P*-value = 0.006, CC = 0.6). Building on these insights, we further extracted and scrutinized a dataset encompassing 1104 genes from the green and red modules (*P*-value < 0.01 and CC > 0.5).

### 3.3 Vital Genes Intersected between DEGs and WGCNA Gene Modules

In this study, we identified 119 vital genes at the intersection of DEGs and WGCNA modules, as shown in Fig. (**3A**). Among these, 29 genes were up-regulated, and 90 were down-regulated, as detailed in Fig. (**3B**). The up-regulated genes were predominantly enriched in GO terms such as regulation of body fluid levels, body fluid secretion, odontogenesis, and multi-organism reproductive process. Furthermore, KEGG pathway enrichment analysis revealed significant associations with Alcoholism, IL-17 signaling pathway, and Relaxin signaling pathway, as illustrated in Fig. (**3C**). Conversely, down-regulated genes showed enrichment in processes like monoatomic anion transport, bicarbonate transport, and one-carbon compound transport, with KEGG enrichment indicating links to Cushing syndrome, Bladder cancer, and GnRH secretion, depicted in Fig. (**3D**). GSEA further elucidated that Regulation of Molecular Function and Animal Organ Morphogenesis is integral in the pathogenesis of microtia, as presented in Fig. (**3E**).

### 3.4 Feature Genes Screened by SVM-RFE and PPI Network

**Fig. (4):**
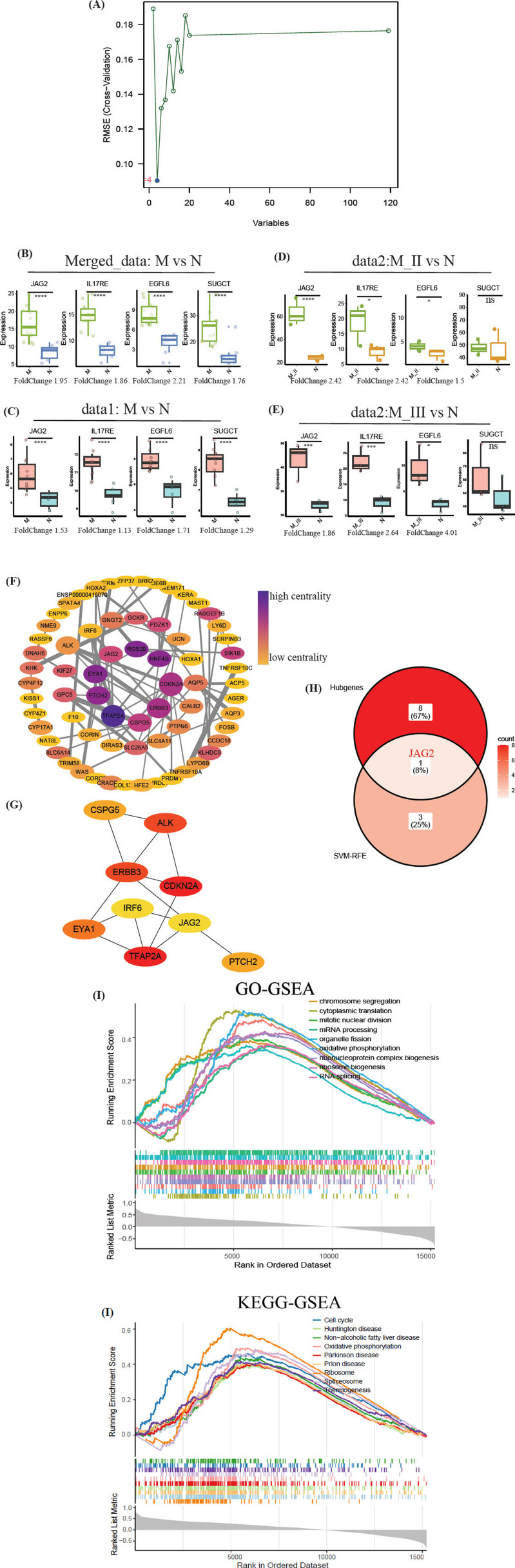
Feature genes screened by the SVM-RFE algorithm and their PPI network. (**A**). SVM-RFE screened 4 feature genes: JAG2, IL17RE, EGFL6 and SUGCT. (**B**). Expression of 4 feature genes in merged data. All of them were up-regulated in the microtia group. (**C**). Verification of gene expression in data1 (GSE225225). (**D-E**). Verification of gene expression in data2 (GSE227119). (**F**). PPI network of proteins translated by vital genes intersected between DEGs and WGCNA gene modules. (**G**). Hub genes identified by ‘cytoHubba’. (**H**). Intersection of hub genes and genes screened by SVM-RFE. (**I**). Single gene GO-GSEA analysis of JAG2. (**J**). Single gene KEGG-GSEA analysis of JAG2. (SVM-RFE: Support Vector Machine-Recursive Feature Elimination; PPI: Protein to Protein Interaction network. *p<0.05, ***p<0.001, ****p<0.0001, ns: no significance).

Utilizing SVM-RFE, we identified four key feature genes: *JAG2, IL17RE, EGFL6,* and *SUGCT*, as shown in Fig. (**4A**) and marked in Fig. (**2C**). Expression and foldchanges of 4 genes in merged_data (Fig. **4B**), data1 (Fig. **4C**), and data2 (Fig. **4D**, **E**) were detected by “DESeq2”. In the PPI network constructed using the 119 vital genes at the intersection of DEGs and WGCNA modules (Fig. **4F**), *JAG2* emerged as a central and pivotal player, highlighted which was identified as one of the hub genes by “cytoHubba” in “Cytotrace” (Fig. **4G**). Consequently, *JAG2* was posited as a potential target biomarker (Fig. **4H**). Further investigation through *JAG2* single gene GO-GSEA analysis indicated significant enrichment in processes like chromosome segregation, cytoplasmic translation, and mitotic nuclear division (Fig. **4I**). Additionally, KEGG-GSEA analysis revealed notable enrichment in pathways including Cell Cycle, Huntington”s Disease, and Non-Alcoholic Fatty Liver Disease (Fig. **4J**).

### 3.5 Verifying Expression of Feature Genes by Single-cell Transcriptome Data, Immunofluorescence and RT-PCR

The single-cell dataset GSE179135, sourced from the GEO database, underwent a series of preprocessing steps. This process led to the classification of all cells into 5 distinct clusters. Of these, we focused on 3 clusters pertinent to chondrocytes: Chond (mature chondrocytes), CPC (chondrocyte progenitor cells), and SC (stromal cells), as depicted in Fig. (**5A**). For detailed classification criteria, readers are referred to the original article [12]. Notably, in this article, the CPC cluster was further subdivided into SSPC (stromal stem/progenitor cells) and CSPC (chondral stem/progenitor cells), both of which exhibited progenitor cell characteristics. Consequently, we consolidated these subtypes into a single-cell category – CPC. A dot plot analysis revealed the expression patterns of the four feature genes across different cell types, as shown in Fig. (**5B**).

Subsequent statistical analysis discerned significant differences in the expression of the 4 feature genes between the microtia and normal groups across the three cell types, detailed in Fig. (**5C**). Notably, *JAG2* exhibited marked up-regulation in the Chond cluster. Expanding on this finding, we assessed *JAG2* expression through PCR (n = 4 samples for each group) and immunofluorescence techniques (n = 29 cells for each group), with results presented in Figs. (**5D** and **5E**), respectively. Both methods corroborated the significant up-regulation of JAG2.

### 3.6 Further Understanding of Microtia Chondrocytes based on Combining Bulk and Single-cell RNA Transcriptome Data

**Fig. (6):**
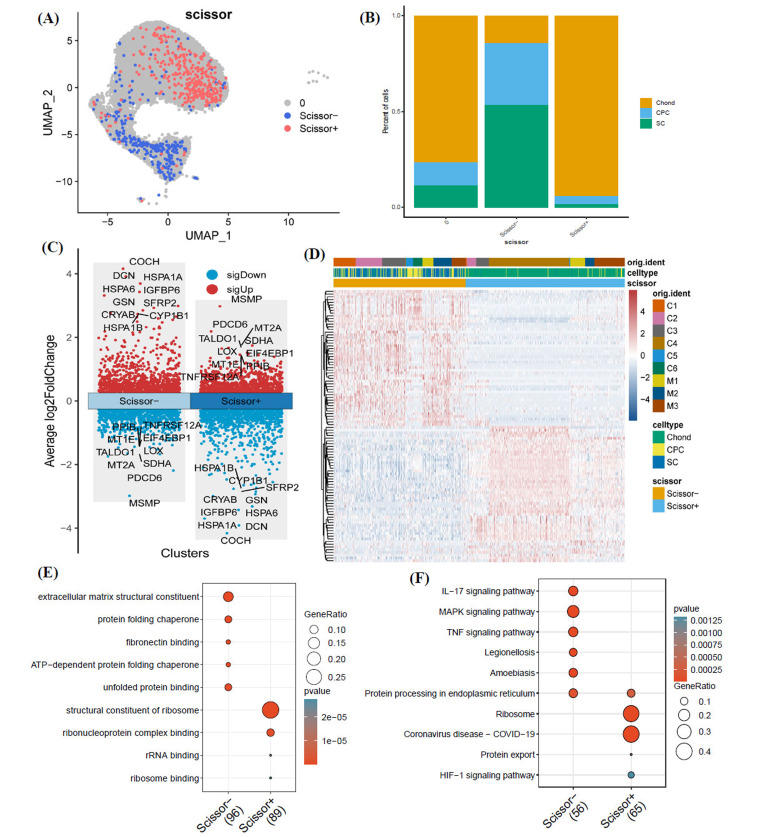
Further understanding of microtia chondrocytes based on combining bulk and single-cell RNA transcriptome data. (**A**). UMAP of scissor types based on combining bulk and single-cell RNA transcriptome data. Scissors+ represents a higher correlation to microtia phenotype. Scissor represents a higher correlation to a normal phenotype. (**B**). Proportion of different cell types in different 18/27 scissor groups. (**C**). Volcano plot of differentially expressed genes of scissor- and scissor+ cells. (**D**). Heat map of top 100 differentially expressed genes of scissor- and scissor+ cells. (**E**). GO enrichment terms of top 100 differentially expressed genes of scissor- and scissor+ cells. (**F**). KEGG pathways of top 100 differentially expressed genes of scissor- and scissor+ cells. (UMAP: Uniform Manifold Approximation and Projection; GO: Gene Ontology; KEGG: Kyoto Encyclopedia of Genes and Genomes). Genes highly expressed in scissor-cells and scissor+ were enriched in distinct GO terms and KEGG pathways.

To further elucidate the cellular subpopulations implicated in microtia malformation at the single-cell level, we utilized the R package “scissor” for partitioning the single-cell data into three distinct subgroups, informed by bulk RNA data. A “scissor” value of 0 indicated a weak correlation. “scissor+” denoted a subgroup positively correlated with the microtia clinical phenotype, while “scissor-” represented a negative correlation. When these subgroups were mapped onto a UMAP plot of cell types, “scissor+” cells predominantly localized within the Chond cluster, whereas “scissor-” cells were mainly observed in the CPC and SC clusters, as seen in Figs. (**6A** and **6B**). The differential expression patterns between scissor- and scissor+ cell types were captured in a volcano plot (Fig. **6C**), and a heatmap showcasing the top expressed genes in these cells is presented in Fig. (**6D**-**F**).

## 4 DISCUSSION

The condition of microtia not only significantly impacts physical appearance but also gives rise to severe psychological and socioeconomic challenges. With the advancement of high-throughput sequencing technology, an increasing abundance of multi-omics sequencing data is available to investigate the pathogenesis of microtia. However, previous studies on microtia malformation sequencing data have predominantly relied on simplistic differential gene analysis using individual datasets with small sample sizes, posing challenges in accurately identifying genuine biological markers [15]. Here, we synthesized two bulk RNA transcriptome datasets, which significantly increased the sample size, and then we further integrated single-cell sequencing data for a comprehensive understanding of the pathogenesis of microtia and to explore its possible biological targets.

Our initial step involved conducting a differential analysis to identify DEGs. As shown in Fig. (**2**), 396 genes were found to be significantly up-regulated, while 273 genes were significantly down-regulated. We then advanced our investigation through the application of WGCNA, which allowed us to explore correlated gene modules. Subsequently, a critical intersection between DEGs and WGCNA gene modules was identified of 119 vital genes. GO and KEGG analysis indicated significant enrichment of these vital genes in several biological processes and diverse pathways. Furthermore, GSEA highlighted the crucial roles of Regulation of Molecular Function and Animal Organ Morphogenesis in the context of microtia. In previous studies, anion transport [24, 25], Alcoholism [26], and IL-17 signaling pathway [27] in synovial fluid have been confirmed to be associated with chondrocyte physiology. Based on those sequencing results and previous studies, we speculated that these enriched terms might be associated with the pathogenesis of microtia.

To further identify feature genes, we employed the SVM-RFE algorithm for further analysis. SVM-RFE, as a method in machine learning, has been widely applied in the construction of prediction models and the identification of key genes [28-30]. This approach facilitated the screening and identification of four key genes: *JAG2, IL17RE, EGFL6,* and *SUGCT*. Furthermore, within the PPI network [31, 32], constructed using the 119 vital genes, *JAG2* emerged as a central or “hub” gene. The expression trend of *JAG2* showed good consistency in both merged and separated datasets. This prominence of JAG2 strongly suggests its potential role as a biomarker for microtia.

The development of single-cell sequencing technology enables us to explore gene expression at the subcellular level [33, 34]. To overcome the insufficient display of cellular heterogeneity of bulk sequencing, we integrated single-cell and bulk sequencing for further analysis. Interestingly, the above 4 feature genes disclosed notable differences across these three cell types. Particularly, *JAG2* was predominantly up-regulated in the Chond cluster of the microtia group. Furthermore, the expression levels of both mRNA and protein were significantly up-regulated in the microtia sample, which was consistent with the observation of single-cell analysis. These results suggested that *JAG2* may play an important role in the pathogenesis of microtia.

Besides, to elucidate which cellular subpopulations are implicated in microtia malformation, the R package “scissor” was used to identify subpopulations within single-cell data that align with phenotypes observed in bulk sequencing in this study [23, 35, 36]. In other words, we leveraged the microtia/normal phenotypes from the merged bulk data to pinpoint phenotype-associated cell populations in the single-cell dataset. We found that scissor+ cells showed a strong positive correlation with microtia phenotype, while scissor- cells exhibited a strong negative correlation. Predominantly, Chond cells constituted the scissor+ population, while CPC and SC-type cells made up the majority of Scissor- cells. Moreover, *JAG2* was primarily up-regulated in the Chond cluster of microtia samples compared with normal samples. These results further confirmed that JAG2 may account for the pathogenesis of microtia and may serve as a biomarker for microtia.

*JAG2*, the gene encoding Jagged2, is a constituent of the Notch ligand family, specifically known as Jagged Canonical Notch Ligand 2 [37]. Notch ligands interact directly with Notch receptors on adjacent cells (in a trans interaction) to initiate the cleavage of the intracellular domain of the Notch receptor (NICD), which then translocates to the nucleus, activating transcription of downstream target genes. Beyond these trans-activating interactions, Notch ligands and receptors situated on the same cell can also form cis-inhibitory interactions [38]. Although JAG2’s role in the development of microtia has not been previously reported, the Notch signaling pathway is known to be involved in chondrocyte hypertrophy and differentiation [39-41]. It may also play an important role in protecting chondrocytes from senescence [42]. Our KEGG enrichment analysis also highlighted the involvement of the Notch signaling pathway in microtia. Therefore, it is plausible that *JAG2* could have a significant impact on the development of microtia, particularly in relation to chondrocyte development. We speculated that considerable upregulation of *JAG2* might cause functional deficit of mature chondrocytes or impair the differentiation of chondrocyte progenitor cells by activating the Notch signaling pathway, and eventually lead to the occurrence of congenital microtia. This process may be mediated by the influence of the JAG2-Notch signaling pathway on the expression of cartilage-specific transcription factors. For instance, SOX9, a key transcription factor for cartilage development [43-45], was reported to be influenced by this signaling pathway [39, 46, 47]. Notch signaling can also affect the binding in the enhancer regions of type II collagen specific to cartilage, thereby influencing its expression [48]. Additionally, the Notch signaling pathway also interacts with signals affecting embryonic development, such as HOX, which is one of the potential mechanisms leading to the occurrence of microtia [49, 50]. Therefore, JAG2 is a potential prenatal diagnostic marker for non-syndromic microtia. Moreover, the residual ear tissue of microtia patients is an important source of primary chondrocytes. Targeting JAG2 to improve cell phenotypes, thereby improving the performance of tissue-engineered regenerated cartilage, may represent another potential application direction.

Certainly, the current results are insufficient to prove that *JAG2* is a pathogenic gene of microtia, and further investigation is still needed. Due to the limited availability of samples, the datasets and the chondrocytes used for our validation primarily originated from Asian adolescents under the age of 18. The applicability of our findings to microtia patients across a broader age range or from other regions of the world remains to be explored. However, integrated analysis of bulk and single-cell RNA transcriptome data in the current study offered distinct perspectives on RNA expression levels and provided more reliable results for future study.

## CONCLUSION

In summary, we conducted a comparative analysis of the RNA transcriptome data from two bulk transcriptome datasets and a single-cell dataset to find out the potential pathogenic or susceptible genes for congenital microtia. Through the utilization of integrative bioinformatics, we find that *JAG2* may play an important role in the pathogenesis of microtia. The up-regulation of *JAG2* may affect the maturation and differentiation of chondrocytes by activating Notch signaling, which eventually leads to the occurrence of microtia. Although the current results are insufficient to fully prove that *JAG2* is a pathogenic gene of microtia and further investigation is still needed, this study provides detailed insight into the pathogenesis of microtia.

## Figures and Tables

**Fig. (1):**
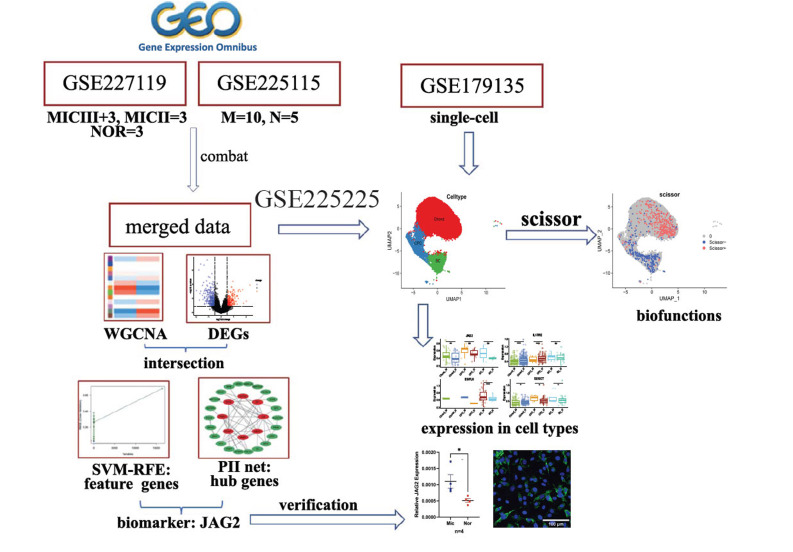
The flowchart of this study.

**Fig. (2):**
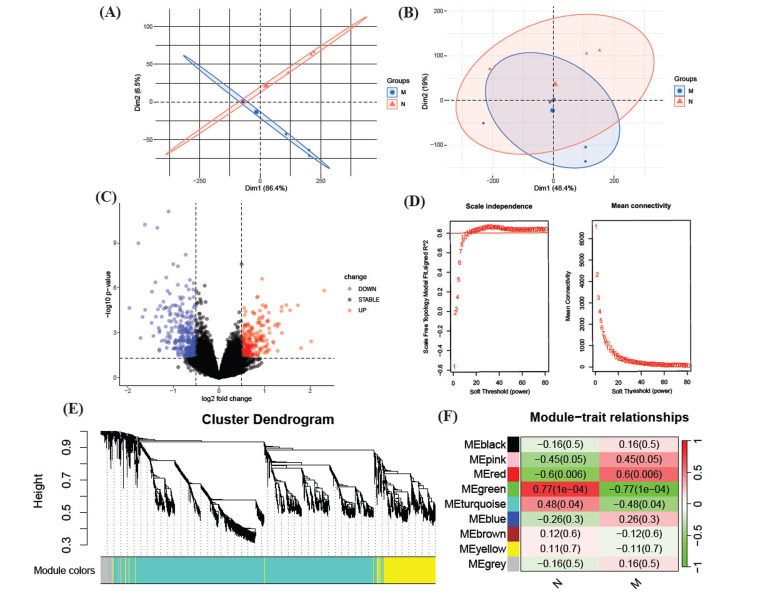
Identifying DEGs and correlated gene modules after merging RNA transcriptome data of two datasets. (**A**). PCA plot of merged data before removing batch effect using the “sva” package. (**B**). PCA plot of merged data after removing batch effect using the “sva” package. The batch effect was clearly removed. (**C**). Volcano plot DEGs identified by R package “DESeq2”. Important feature genes were marked. (**D**). Identification of soft-threshold power by analyzing the scale-free index (left) and the mean connectivity (right) in the WGCNA. (**E**). Dendrogram of all DEGs clustered based on a dissimilarity measure(1-TOM). Clustered genes are shown in colors. (**F**). Heatmap showing the correlation between ME and phenotype. The CC and *P*-values of each module in microtia and normal 10/27 group are presented in the center of the panels. The closer of the CC is to 1, the higher its relevance is. Green and red modules reveal a significant correlation to microtia. (PCA: Principal Component Analysis; DEG: Differentially Expressed Gene; WGCNA: Weighted Gene Co-expression Network Analysis; CC: Correlation Coefficient, TOM: Topological Overlap Matrix; ME: Module Eigengene; M: Microtia; N: Normal).

**Fig. (3):**
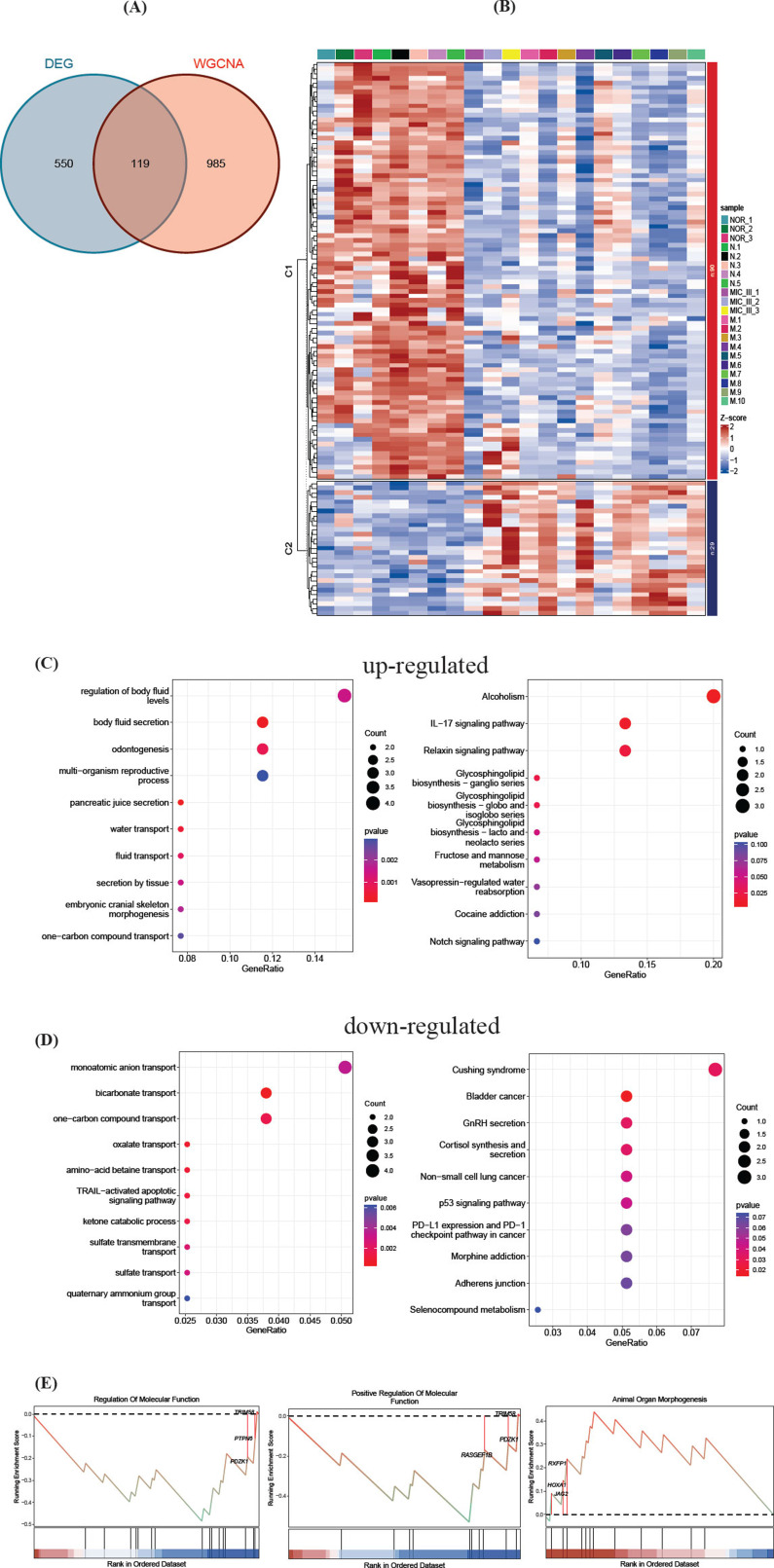
Vital genes intersected between DEGs and correlated gene modules. (**A**). Venn diagram shows the intersection of DEGs and WGCNA. One hundred nineteen genes were intersected between DEGs and WGCNA. (**B**). Heat map of vital intersected genes. Compared to the normal group, the C1 gene set contains 90 downregulated genes, and C2 contains 29 upregulated genes. (**C**). GO and KEGG enrichment terms of upregulated genes. (**D**). GO and KEGG enrichment terms of downregulated 12/27 genes. (**E**). Top 3 bioprocesses of GSEA analysis on vital intersected genes. (GO: Gene Ontology; KEGG: Kyoto Encyclopedia of Genes and Genomes; GSEA: Gene Set Enrichment Analysis).

**Fig. (5):**
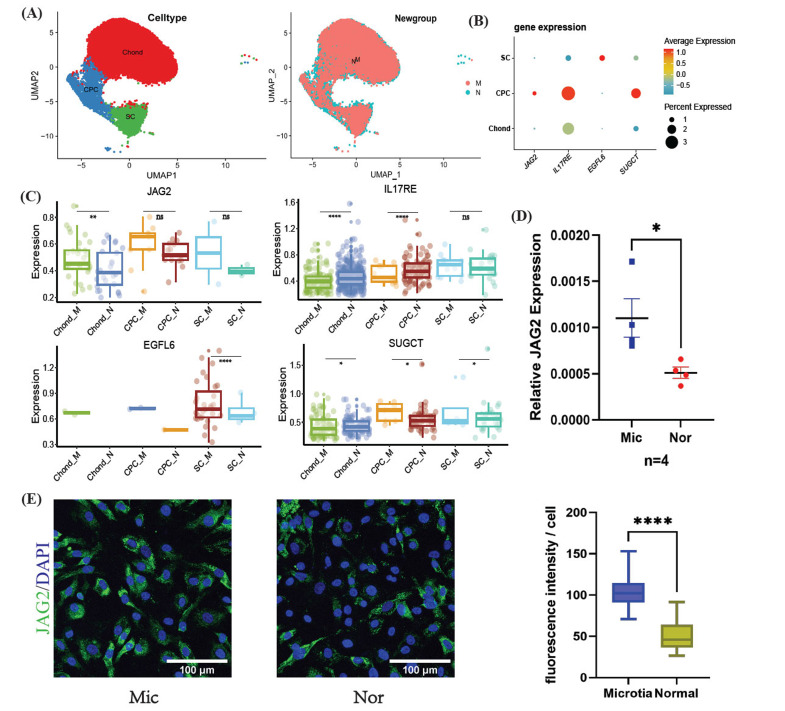
Verification of gene expression by single-cell transcriptome data, immunofluorescence and RT-PCR. (**A**). UMAP of cell types in single-cell data of microtia and normal chondrocytes. (**B**). Overall expression of 4 feature genes in single-cell data. (**C**). Expression difference of 4 feature genes between microtia and normal group in different cell types. JAG2 was significantly up-regulated in the Chond of microtia group compared to the normal group. (**D**). RNA expression difference of JAG2 by RT-PCR. (**E**). JAG2 immunofluorescence in microtia and normal chondrocytes. They both showed similar results. Kruskal–Wallis test was used for all statistical significance. (UMAP: Uniform Manifold Approximation and Projection; PCR: Polymerase Chain Reaction. *p<0.05, **p<0.01, ****p<0.0001, ns: no 16/27 significance).

**Table 1:** Demographic characteristics.

**Samples**	**Diagnosis**	**Age (Year)**	**Nationality**
Patient 1	Microtia III	9	China
Patient 2	Microtia III	9	China
Patient 3	Microtia III	10	China
Patient 4	Microtia III	12	China
Patient 5	Normal	8	China
Patient 6	Normal	16	China
Patient 7	Normal	13	China
Patient 8	Normal	17	China

## Data Availability

GSE227119 and GSE179125 were obtained from the GEO database. GSE225225 was obtained from the corresponding author of GSE225225 in the GEO database.

## LIST OF ABBREVIATIONS

CC: Correlation Coefficient
Chond: Mature Chondrocytes
CPC: Chondrocyte Progenitor Cells
DEG: Differentially Expressed Gene
GO: Gene Ontology
GSEA: Gene Set Enrichment Analysis
KEGG: Kyoto Encyclopedia of Genes and Genomes
M: Microtia
ME: Module Eigengene
N: Normal
PCA: Principal Component Analysis
PPI: Protein to Protein Interaction
Rt-PCR: Real-Time Polymerase Chain Reaction
SC: Stromal Cells
SVM-RFE: Support Vector Machine-Recursive Feature Elimination
TOM: Topological Overlap Matrix
UMAP: Uniform Manifold Approximation and Projection
WGCNA: Weighted Gene Co-expression Network Analysis
